# mRNA Secondary Structures Fold Sequentially But Exchange Rapidly In Vivo

**DOI:** 10.1371/journal.pbio.1000307

**Published:** 2010-02-09

**Authors:** Elisabeth M. Mahen, Peter Y. Watson, Joseph W. Cottrell, Martha J. Fedor

**Affiliations:** 1 Department of Molecular Biology, The Scripps Research Institute, La Jolla, California, United States of America; 2 The Skaggs Institute for Chemical Biology, The Scripps Research Institute, La Jolla, California, United States of America; 3 Department of Chemical Physiology, The Scripps Research Institute, La Jolla, California, United States of America; University of Wisconsin, United States of America

## Abstract

RNAs adopt defined structures to perform biological activities, and conformational transitions among alternative structures are critical to virtually all RNA-mediated processes ranging from metabolite-activation of bacterial riboswitches to pre-mRNA splicing and viral replication in eukaryotes. Mechanistic analysis of an RNA folding reaction in a biological context is challenging because many steps usually intervene between assembly of a functional RNA structure and execution of a biological function. We developed a system to probe mechanisms of secondary structure folding and exchange directly in vivo using self-cleavage to monitor competition between mutually exclusive structures that promote or inhibit ribozyme assembly. In previous work, upstream structures were more effective than downstream structures in blocking ribozyme assembly during transcription in vitro, consistent with a sequential folding mechanism. However, upstream and downstream structures blocked ribozyme assembly equally well in vivo, suggesting that intracellular folding outcomes reflect thermodynamic equilibration or that annealing of contiguous sequences is favored kinetically. We have extended these studies to learn when, if ever, thermodynamic stability becomes an impediment to rapid equilibration among alternative RNA structures in vivo. We find that a narrow thermodynamic threshold determines whether kinetics or thermodynamics govern RNA folding outcomes in vivo. mRNA secondary structures fold sequentially in vivo, but exchange between adjacent secondary structures is much faster in vivo than it is in vitro. Previous work showed that simple base-paired RNA helices dissociate at similar rates in vivo and in vitro so exchange between adjacent structures must occur through a different mechanism, one that likely involves facilitation of branch migration by proteins associated with nascent transcripts.

## Abstract 2

Self-cleavage assays of RNA folding reveal that mRNA structures fold sequentially in vitro and in vivo, but exchange between adjacent structures is much faster in vivo than it is in vitro.

## Author Summary

Properly folded RNAs are critical for virtually all RNA-mediated processes ranging from feedback regulation of gene expression to RNA maturation. The ability of RNAs to adopt specific structures in living cells is remarkable given their propensity to become trapped in a mixture of stable, misfolded structures in vitro. Using mRNA with an inserted ribozyme and self-cleavage to monitor competition between mutually exclusive structures, we previously showed that upstream structures dominated folding outcomes during RNA synthesis in vitro, suggesting that folding occurs sequentially. However, when studied in vivo upstream and downstream structures blocked ribozyme assembly equally well in yeast, providing evidence that intracellular folding outcomes reflect the relative stability of alternative structures. We find that very stable upstream structures can block assembly of downstream structures in vivo even when the downstream structures are more stable, and that a narrow threshold of stability determines whether folding and unfolding rates or thermodynamic stability govern folding outcomes. Thus, mRNAs fold sequentially in vitro and in vivo but exchange between adjacent structures is faster in vivo than in vitro. Simple RNA structures unfold at similar rates in vivo and in vitro, so exchange between adjacent structures in vivo probably occurs through a distinct, step-wise mechanism that could be facilitated by proteins associated with nascent RNAs.

## Introduction

**Figure 1:**
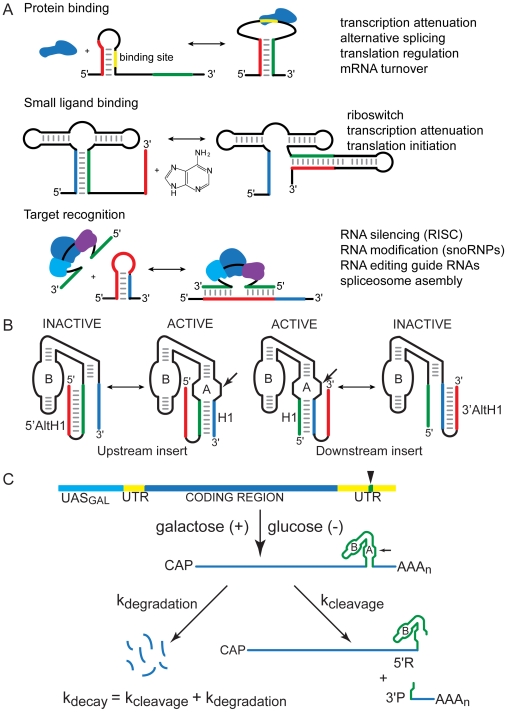
RNA secondary structure folding and exchange. (A) Exchange among alternative RNA secondary structures plays essential roles in virtually all RNA-mediated processes including transcription and translation regulation, precursor RNA maturation, and RNA interference. (B) Chimeric mRNA self-cleavage reflects competition between two mutually exclusive structures: an H1 helix (blue and green) needed for assembly of a functional ribozyme and a nonfunctional AltH1 stem-loop (red and green). (C) Quantitative analysis of RNA folding in vivo. HP sequences (green) are inserted into the 3′ UTR (yellow) of the yeast *PGK1* gene and transcribed under the control of the GAL1-10 upstream activation sequence, UAS_GAL_ (aqua), to allow measurement of HP mRNA decay kinetics after glucose inhibition. HP mRNA decays both through self-cleavage (k_cleavage_) and through the normal mRNA degradation pathway (k_degradation_), so self-cleavage accelerates HP mRNA decay by an amount that corresponds to the intracellular cleavage rate.

RNAs adopt specific secondary structures to carry out their biological functions, and exchange among alternative secondary structures plays essential roles in virtually all RNA-mediated processes ranging from RNA silencing and metabolite-activation of bacterial riboswitches to pre-mRNA splicing and viral RNA replication (Figure 1A) [1]–[6]. The ability of RNAs to assemble into precise structures and undergo transitions from one defined structure to another on a biological time scale is remarkable since RNAs tend to adopt a mix of misfolded structures with slow exchange kinetics in vitro [7]–[9]. Thus, detailed understanding of the mechanisms of RNA assembly and exchange as it occurs in vivo is critical for understanding RNA function.

Two kinds of mechanisms have been proposed to explain how RNA secondary structures form and assemble into tertiary structures precisely and efficiently in vivo. First, RNA chaperones might facilitate thermodynamic equilibration by lowering the free energy barrier for RNA unfolding and refolding [10]–[14]. Many proteins, particularly basic unstructured proteins, exhibit general RNA chaperone activity in vitro [15]–[17]. The DEAD-box family of putative RNA helicases has been implicated in virtually every aspect of RNA metabolism including ribosome biogenesis, pre-mRNA splicing, RNA interference, translation, mRNA transport, and decay [18]–[20]. Although they exhibit little substrate specificity in vitro, most DEAD-box proteins function as part of a large macromolecular complex, such as a spliceosome or degradosome, that is devoted to a particular process. Although certain DEAD-box proteins have been shown to facilitate self-splicing and translation of a variety of RNAs in vivo and in vitro [21],[22], it is not yet clear whether nonspecific chaperones act generally to promote assembly of RNAs into functional structures or accelerate exchange among alternative structures in vivo. Second, the sequence and timing with which regions of a nascent RNA become available to fold during transcription also might channel it into a productive folding pathway. RNA secondary structure folding occurs on a microsecond time scale [23],[24], a rate that is much faster than elongation by RNA polymerase II, which transcribes at a rate of about 100 nucleotides per second in vivo [25]. Therefore, folding of a nascent transcript as it emerges from the polymerase could favor local secondary structures and limit long-range interactions. Evidence that elongation kinetics, transcriptional pausing, and circular permutations of RNA sequences influence folding patterns support the idea that RNA secondary structures fold sequentially in vivo [26]–[30].

Probing RNA folding mechanisms in a biological context is challenging because many components interact in complex pathways and several steps usually intervene between assembly of an RNA structure and execution of a biological function. We developed a system to investigate intracellular RNA folding that relies on hairpin ribozyme (HP) cleavage kinetics to report directly and quantitatively on partitioning between two mutually exclusive RNA secondary structures, helix 1 (H1) and alternative helix 1 (AltH1), in chimeric mRNAs (Figure 1B) [31]–[33]. The 3′ untranslated region (UTR) of a chimeric mRNA contains a self-cleaving ribozyme sequence and a complementary sequence, located either upstream or downstream of the ribozyme, that has the potential to anneal with part of the ribozyme sequence and block formation of the H1 helix needed for assembly of a functional ribozyme. Thus, part of the ribozyme sequence can participate in one of two mutually exclusive base-paired structures, similar to the kinds of RNA conformational switches that have been implicated in biological regulation of RNA silencing, pre-mRNA splicing, mRNA turnover, viral genome replication, translation initiation, transcription attenuation, and in metabolite-triggering of bacterial riboswitches, as examples [1]–[6]. The alternative secondary structures formed by these chimeric RNAs were designed to have well-defined structures and thermodynamic stabilities that facilitate quantitative analyses, but yeast do not normally have RNAs like HPs and are not likely to contain any ribozyme-specific ligands. Therefore, the behavior of these chimeric mRNAs should reflect general features of RNA folding in an intracellular environment. Differences in the folding behavior of chimeric RNAs with inserts located upstream or downstream of the ribozyme reflect the influence of 5′ to 3′ transcriptional polarity, and the behavior of RNAs with different H1 and AltH1 structures reflects the influence of folding and unfolding kinetics and thermodynamic stability on folding outcomes.

Self-cleaving RNAs are expressed in yeast as chimeric mRNAs under the control of a glucose-repressible promoter that enables quantification of intracellular RNA turnover rates (Figure 1C). Chimeric mRNAs that assemble into functional ribozyme structures decay through self-cleavage and through endogenous mRNA degradation pathways while chimeric mRNAs with mutationally inactivated ribozymes decay only through endogenous degradation pathways. Therefore, the difference between intracellular decay rates for mutant and self-cleaving mRNAs reflects partitioning between the H1 helix of a functional ribozyme and nonfunctional AltH1 structures.

We previously examined chimeric RNAs with the potential to form an H1 helix with eight base pairs in competition with AltH1 helices with 10 base pairs that have greater thermodynamic stability (Figure 1B) [32]. Complementary inserts located upstream of the ribozyme inhibited ribozyme assembly more than downstream inserts during transcription in vitro, consistent with a sequential folding mechanism in which a stable structure that forms first dominates the folding outcome. These H1 and AltH1 structures with eight or 10 base pairs have sufficiently high thermodynamic stability that they are not expected to dissociate for months, or even years, under standard conditions in vitro [31],[34]–[37]. Therefore, it was not surprising that a stable upstream 5′ AltH1 could prevent H1 folding from a downstream sequence that was not transcribed until after the 5′ AltH1 had formed. When the same variants were expressed as chimeric mRNAs in yeast, however, upstream and downstream inserts blocked ribozyme assembly equally well. The ability of a downstream 3′ AltH1 structure to interfere with assembly of an upstream ribozyme that can fold first suggested that structures that are kinetically stable in vitro undergo rapid equilibration in vivo and allow intracellular folding to reach thermodynamic equilibrium or that AltH1 folding from contiguous sequences had a kinetic advantage over H1 folding from separate ends of the ribozyme.

We have extended these studies to learn when, if ever, thermodynamic stability becomes an impediment to exchange between alternative RNA secondary structures in vivo. We found that stable upstream structures can block folding of downstream structures in vivo even when downstream structures have greater thermodynamic stability, consistent with a sequential folding mechanism. However, the thermodynamic stability needed to inhibit exchange was much greater in vivo than in vitro. In contrast, the simple helix dissociation reactions required for cleavage product release occur at virtually the same rates in vivo and in vitro [36],[38]. Differences between slow rates of simple helix dissociation and rapid exchange between adjacent secondary structures with moderate stability might be explained by the ability of proteins associated with nascent transcripts to facilitate branch migration.

## Results

### Stable Upstream Structures Resist Competition from Downstream Structures In Vitro and In Vivo

**Figure 2:**
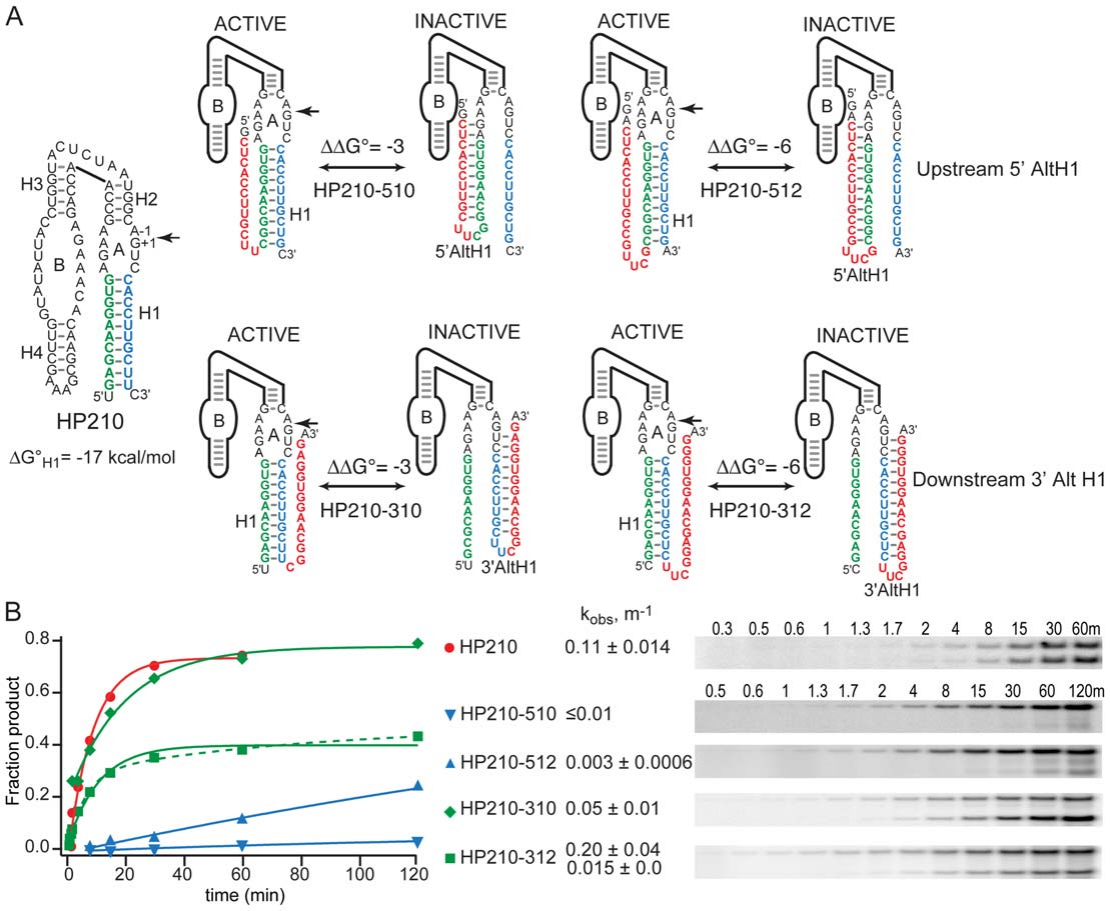
Competition between folding of functional ribozymes and downstream AltH1 structures with greater thermodynamic stability in vitro. (A) HP variants designed to probe RNA secondary structure folding and exchange mechanisms. Sequences of HPs with the potential to fold into a functional ribozyme by annealing of ribozyme sequences (blue and green) to form the essential H1 helix or by annealing of ribozyme sequences with complementary inserts (red) located upstream or downstream of the ribozyme to form nonfunctional 5′ AltH1 or 3′ AltH1 structures, respectively. The unmodified HP210 ribozyme has 10 base pairs in H1 (ΔG°_30°C,H1,calc_ = 17 kcal/mol). Ribozyme variants with 10 or 12 complementary nucleotides inserted upstream (HP210-510 and HP510-512) or downstream (HP210-310 and HP210-312) of the ribozyme have the potential to form 10-base-pair or 12-base-pair AltH1 structures that are more stable than the H1 structures by 3 or 6 kcal/mol, respectively. (B) Self-cleavage activity reflects partitioning between folding of functional ribozymes and competing stem-loop structures during transcription in vitro. Solid lines represent fits to a single exponential rate equation. The dashed line represents the fit of HP210-312 data to a double exponential rate equation that gave two k_obs_ values with nearly equal amplitudes. Plots display results from a single representative experiment. Reported values represent the mean and standard deviation obtained from two or more experiments.

In order to determine whether thermodynamic stability ever becomes an impediment to secondary structure exchange in vivo, we systematically increased the thermodynamic stabilities of competing H1 and AltH1 structures relative to the structures that exchanged freely in our previous study. We began by adding two base pairs to H1, increasing its length from eight to 10 base pairs to create HP210 (Figure 2A). Addition of two base pairs enhances H1 stability by about 2 kcal/mol [35],[37], a change that is expected to slow dissociation of this H1 helix by more than 20-fold relative to the H1 helix with eight base pairs that we examined previously (Table 1). Complementary inserts located upstream of the ribozyme can anneal with the 5′ strand of H1 to form 5′ AltH1 stem loops with 10 base pairs, in HP210-510, or 12 base pairs, in HP210-512. Likewise, complementary inserts located downstream of the ribozyme can anneal with the 3′ strand of H1 to form 3′ AltH1 stem loops with 10 base pairs, in HP210-310, or 12 base pairs, in HP210-312. The H1 helices with 10 base pairs still have lower thermodynamic stability than AltH1 structures with 10 or 12 base pairs by 3 or 6 kcal/mol, respectively [35],[37].

**Table 1:** Thermodynamic and kinetic parameters of alternative secondary structure folding.

	ΔG°_30°C,calc_ [35],[37] kcal/mol				
RNA	H1	5′ AltH1	3′ AltH1	ΔΔG°_30°C,calc_	In Vivo k_cleav_ Min^−1^
HP28 [32]	−15.2				0.034±0.01
HP28-59 [32]	−15.2	−15.1		+0.1	0.052±0.014
HP28-39 [32]	−15.2		−15.7	−0.5	0.032±0.008
HP28-510 [32]	−14.8	−19.4		−4.6	<0.004
HP28-310 [32]	−14.7		−21.3	−6.6	<0.004
HP210	−17.1				0.082±0.007
HP210-510	−17.2	−20.2		−3.0	<0.004
HP210-310	−17.3		−20.5	−3.2	0.049±0.007
HP210-512	−18.7	−24.5		−5.8	<0.004
HP210-312	−18.7		−24.5	−5.8	0.021±0.008
HP214	−28.2				0.066±0.01
HP214-512	−28.2	−25.0		+3.2	≤0.005
HP214-312	−28.2		−25.0	+3.2	0.054±0.03
HPC28	−15.3				0.036±0.008
HPC28-510	−15.3	−20.5		−5.2	0.021±0.006
HPC28-310	−15.3		−20.6	−5.3	0.022±0.004

Ribozyme variants with upstream or downstream inserts displayed very different self-cleavage activity during co-transcriptional assembly in vitro (Figure 2B). Upstream inserts with the potential to form 5′ AltH1 structures with 10 or 12 base pairs inhibited ribozyme assembly and self-cleavage almost completely. In contrast, a downstream insert with the potential to form a 3′ AltH1 structure with 10 base pairs reduced self-cleavage rates only 2-fold. Chimeric RNAs with the potential to form a downstream 3′ AltH1 structure with 12 base pairs partitioned almost equally between fully functional H1 and inactive AltH1 structures. Thus, transcription polarity influenced folding outcomes during co-transcriptional folding in vitro, consistent with the sequential mechanism of secondary structure assembly that we inferred from previous results [32].

**Figure 3:**
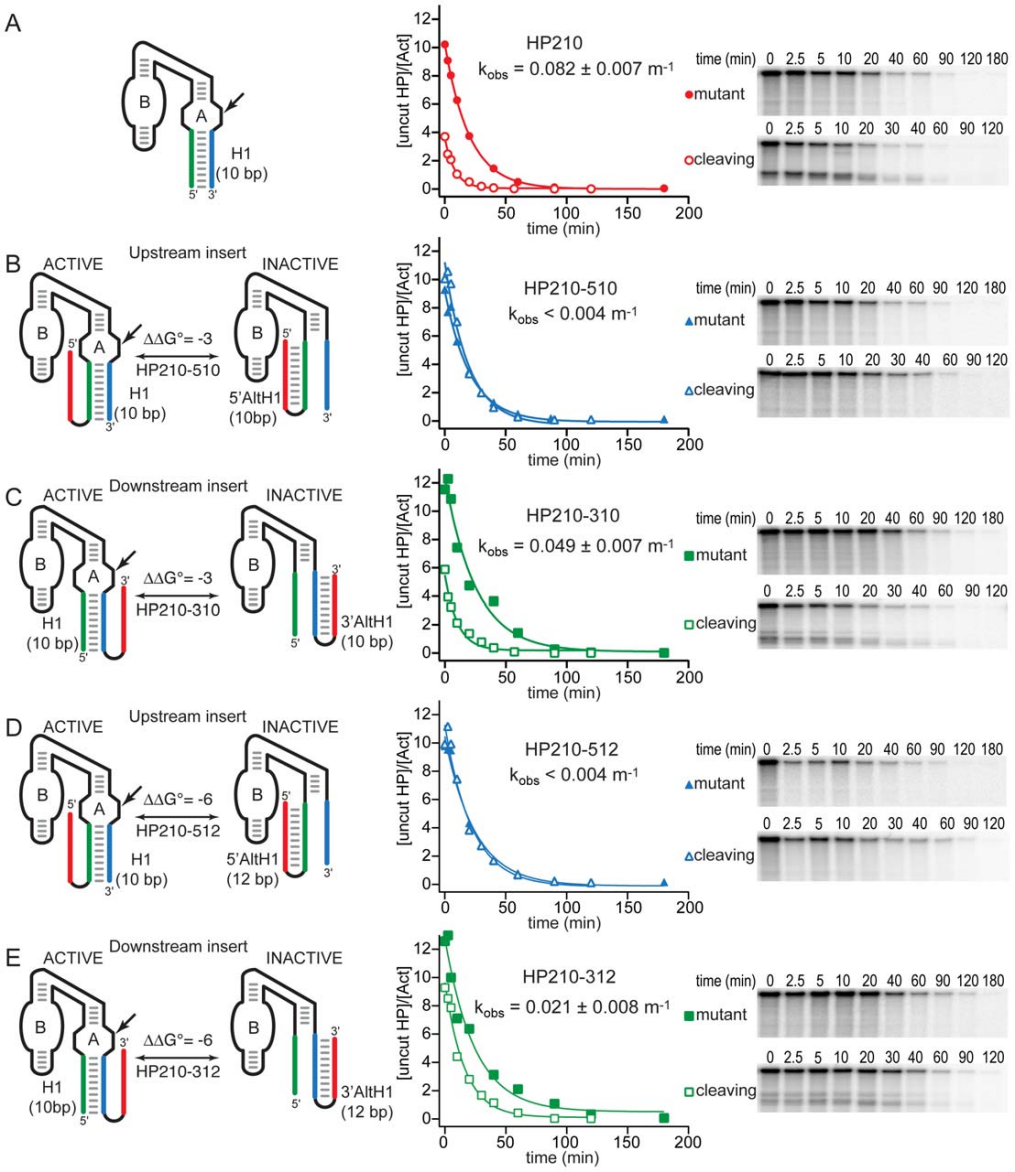
Competition between folding of functional ribozymes and downstream AltH1 structures with greater thermodynamic stability in vivo. Chimeric HP mRNAs were expressed in yeast as chimeric *PGK1* mRNAs subject to glucose inhibition, as described in Figure 1C. k_obs_ values represent assembly of functional ribozyme structures determined from the difference between decay rates measured for self-cleaving mRNAs (k_cleavage_ + k_degradation_) and decay rates measured for mutationally activated chimeric mRNAs that decay only through the normal mRNA degradation pathway (k_degradation_). (A) Unmodified ribozyme with 10 base pairs in H1. (B–E) Ribozymes with the potential to form alternative nonfunctional secondary structures, AltH1, that have greater thermodynamic stability than the essential H1 helix of the ribozyme and are located upstream or downstream of the ribozyme sequence. Assembly of functional ribozymes despite competition from downstream 3′ AltH1 structures that have greater thermodynamic stability (C and E) is consistent with sequential folding of RNA secondary structures in vivo. Plots display results from a representative pair of experiments with functional and mutationally inactivated chimeric mRNAs. Reported values represent the mean and standard deviation obtained from two or more pairs of experiments.

In yeast, the two-base-pair extension of H1 did not rescue ribozyme assembly in chimeric mRNAs with an upstream insert capable of forming a 5′ AltH1 structure with 10 base pairs. That is, HP210-510 still displayed no acceleration of intracellular decay kinetics relative to its mutationally inactivated counterpart, and no cleavage product RNAs were detected in RNase protection assays (Figure 3B, Table 1). However, competition between H1 with 10 base pairs and a downstream 3′ AltH1 structure with 10 base pairs in HP210-310 mRNA had a very different outcome in vivo than we observed previously when H1 contained only eight base pairs [32]. HP210-310 mRNA decayed faster than its mutationally inactivated counterpart, indicating that intracellular cleavage combined with normal mRNA degradation to accelerate intracellular decay kinetics, and RNase protection assays revealed intracellular cleavage products (Figure 3C, Table 1). The nonfunctional AltH1 structures in HP210-510 and HP210-310 RNAs are expected to dominate the folding outcome by about 150-fold relative to functional H1 structures in a rapid equilibration mechanism in which secondary structure folding reaches thermodynamic equilibrium. However, the intracellular cleavage rate of 0.049 min^−1^ calculated for HP210-310 mRNA was only 2-fold lower than the rate of 0.082 min^−1^ measured for the HP210 RNA that lacked any complementary insert. The ability of an upstream H1 helix with 10 base pairs to dominate the folding outcome, even when the alternative downstream structure has greater thermodynamic stability, suggests that secondary structures formed sequentially both in vitro and in vivo.

It was important to confirm that this change in intracellular secondary structure partitioning resulted from the increased kinetic stability of H1 and not from inaccuracy in the free energy calculations that indicated that the H1 helix was less stable than the 3′ AltH1 structure in HP213-310 RNA. HP210-512 and HP210-312 RNAs have the same H1 sequence as HP210-510 and HP210-310 RNAs, but they have two additional base pairs in the AltH1 stem loops that are expected to lower the AltH1 free energy by 3 kcal/mol (Figure 2A). In these variants, the H1 helices were calculated to be less stable than the AltH1 helices by 6 kcal/mol. With a thermodynamic advantage of 6 kcal/mol, nonfunctional AltH1 structures with 12 base pairs would dominate the folding outcome by more than 10^4^-fold relative to functional H1 structures with 10 base pairs if folding reaches thermodynamic equilibrium.

An upstream insert able to form a 5′ AltH1 structure with 12 base pairs inhibited assembly of a functional ribozyme much more than a downstream insert during co-transcriptional assembly of HP210-512 RNA in vitro, as we previously observed for HP210-510 RNA with 10-base-pair 5′ AltH1 structures (Figure 2B) [32]. A chimeric mRNA with 12 base pairs in an upstream 5′ AltH1 stem loop also exhibited no detectable self-cleavage activity in yeast, evidence that folding of a stable, upstream 5′ AltH1 dominated the folding outcome as expected (Figure 3D, Table 1). However, chimeric mRNA with a downstream insert capable of forming a 3′ AltH1 with 12 base pairs displayed an intracellular cleavage rate that was reduced only 4-fold relative to chimeric HP210 mRNA with no insert (Figure 3E, Table 1). The resistance of H1 sequences in each of these HP210 variants to chemical modification by dimethyl sulfate (DMS) in vivo suggests that chimeric mRNAs with the potential to form 10 base pairs in H1 adopt functional ribozyme structures in vivo, consistent with the activity observed in functional assays (Figures S1 and S2). The ability of HP210-310 and HP210-312 mRNAs with 10-base-pair H1 helices to resist competition from a downstream 3′ AltH1 structure that has 10 or 12 base pairs supports the conclusion that the two additional base pairs added to H1 rescued intracellular self-cleavage activity by slowing exchange between functional and nonfunctional structures.

**Figure 4:**
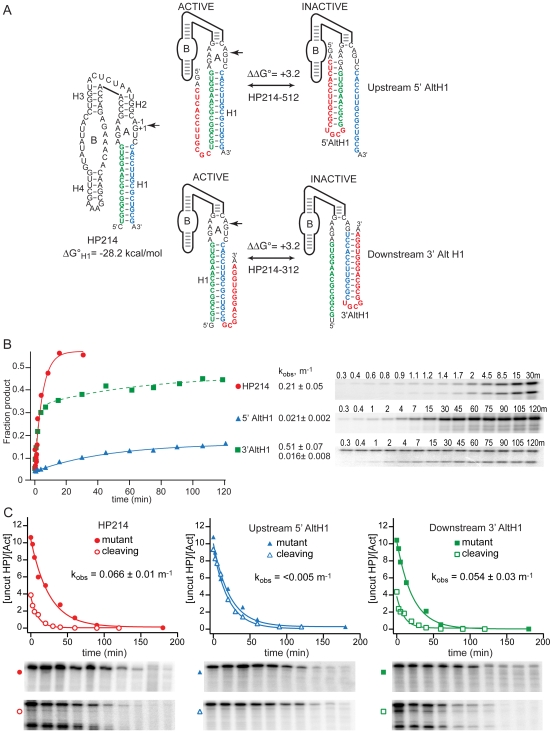
Competition between folding of H1 with 14 base pairs and nonfunctional AltH1 structures with lower thermodynamic stability. (A) Sequences of HPs with the potential to fold into a functional ribozyme by annealing of ribozyme sequences shown in blue and green to form the essential H1 helix or to form nonfunctional AltH1 structures by annealing of the ribozyme sequences with complementary insertions (red) located upstream or downstream of the ribozyme to form nonfunctional 5′ AltH1 or 3′ structures, respectively. H1 structures with 14 base pairs (ΔG°_30°C,helix,calc_ = 28.2 kcal/mol) are more stable than AltH1 structures with 12 base pairs by 3.2 kcal/mol). (B) Self-cleavage activity reflects partitioning between assembly of functional ribozymes and competing stem-loop structures during transcription in vitro. Solid lines represent fits to a single exponential rate equation. The dashed line represents the fit of HP214-312 data to a double exponential rate equation that gave high and low k_obs_ values with amplitudes of approximately 0.13 and 0.3, respectively. Plots display results from a single representative experiment. Reported values represent the mean and standard deviation obtained from two or more experiments. (C) Competition between assembly of functional ribozymes and downstream AltH1 structures with lower thermodynamic stability. Inhibition of assembly of functional ribozymes by an upstream 5′ AltH1 structure that has lower thermodynamic stability (HP214-512) is consistent with sequential folding of RNA secondary structures in vivo. Plots display results from a representative pair of experiments with functional and mutationally inactivated chimeric mRNAs. Reported values represent the mean and standard deviation obtained from two or more pairs of experiments.

In the third series of variants, both H1 and AltH1 helices are much more stable than the secondary structures in the chimeric RNAs examined previously (Table 1). HP214-512 and HP214-312 RNAs have the potential to form 14 base pairs in H1 and 12 base pairs in AltH1 (Figure 4A). Free energy calculations indicate that H1 is more stable than 5′ AltH1 and 3′ AltH1 by 3.2 kcal/mol so the functional ribozyme structure is expected to dominate the folding outcome by more than 200-fold if secondary structure assembly reaches thermodynamic equilibrium. HP214-512, in which 5′ AltH1 forms from upstream sequences, displayed very little cleavage activity during co-transcriptional folding in vitro while a large fraction of HP214-312, the variant with the potential to form a downstream 3′ AltH1, assembled into a functional ribozyme (Figure 4B). This pattern is consistent with a sequential mechanism of secondary structure folding, as observed for other chimeric RNAs with stable AltH1 structures during co-transcriptional folding in vitro (Table 1).

Chimeric HP214-512 mRNA, with an upstream insert capable of forming a 5′ AltH1 structure with 12 base pairs, exhibited no detectable intracellular cleavage activity (Figure 4C). HP214-512 mRNA decayed at the same rate as its mutationally inactivated counterpart, and no products of intracellular cleavage were detected in RNase protection assays. Thus, HP214-512 mRNA appeared to fold exclusively into a nonfunctional 5′ AltH1 structure despite the potential to form a downstream H1 helix with greater thermodynamic stability, an interpretation supported by the susceptibility of a 5′ AltH1 loop nucleotide to DMS modification (Figure S3). In contrast, a downstream insert that had the potential to form a 3′ AltH1 structure with 12 base pairs had virtually no inhibitory effect on the ability of chimeric mRNA to form a functional ribozyme structure in vivo. Chimeric HP214-312 mRNA exhibited virtually the same intracellular decay kinetics as HP214 mRNA that lacks a complementary insert (Figure 4C). Furthermore, the 5′ strand of H1 in HP214-312 mRNA is relatively resistant to chemical modification, consistent with the conclusion that HP214-312 mRNA adopts a functional ribozyme structure in vivo (Figure S3). These results are consistent with a sequential folding mechanism in which the H1 helix folds first and does not exchange with a downstream 3′ AltH1 structure that has lower thermodynamic stability.

### Circular Permutation Alters Secondary Structure Partitioning

**Figure 5:**
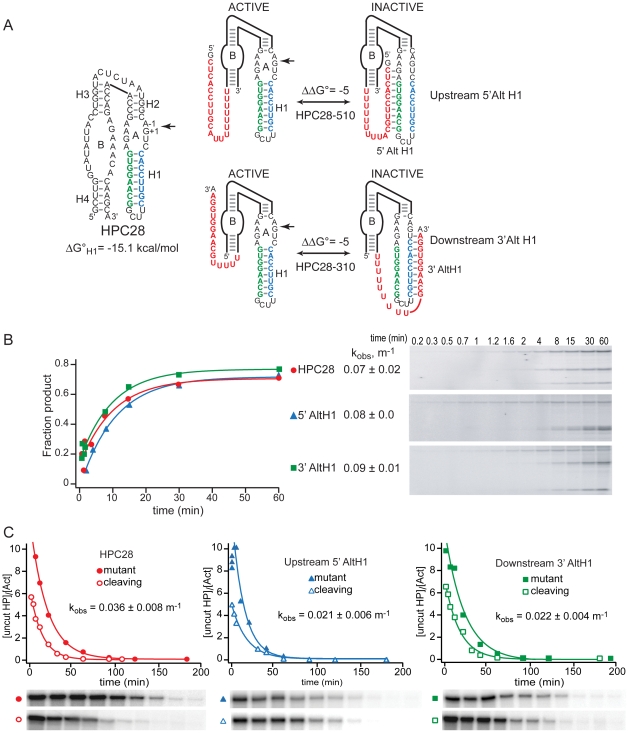
Circular permutations affect partitioning among alternative secondary structures. (A) Sequences of HPs with the potential to fold into a functional ribozyme by annealing of ribozyme sequences shown in blue and green to form the essential H1 helix or to form nonfunctional AltH1 structures by annealing of the ribozyme sequences with complementary insertions (red) located upstream or downstream of the ribozyme to form nonfunctional 5′ AltH1 or 3′ AltH1 structures, respectively, with greater thermodynamic stability. In these ribozyme variants, H1 folds from contiguous sequences in contrast with ribozyme variants studied previously in which AltH1 structures folded from contiguous sequences and functional ribozymes required H1 helices to fold from separate regions. The thermodynamic stabilities calculated for these H1 helices include a contribution of 2 kcal/mol by the stable UNGG tetraloop [40]. The unmodified HPC28 ribozyme and variants with upstream (HPC28-510) or downstream (HPC28-310) insertions display similar assembly and self-cleavage kinetics during transcription in vitro (B) and in vivo (C). Thus, H1 helices that fold rapidly from contiguous sequences resist competition from structures that have greater thermodynamic stability but require interactions with more distal sequences. Lines represent fits to a single exponential rate equation. Plots in (B) display results from a single representative experiment. Reported values represent the mean and standard deviation obtained from two or more experiments. Plots in (C) display results from a representative pair of experiments with functional and mutationally inactivated chimeric mRNAs. Reported values represent the mean and standard deviation obtained from two or more pairs of experiments.

Secondary structure folding from contiguous strands to form AltH1 stem loops is expected to be faster than H1 folding from noncontiguous regions of the RNA because folding rates decrease with increasing loop size [39]. To probe how topology and folding kinetics affect partitioning between alternative structures, we examined circularly permuted ribozymes in which H1 stem loops fold from adjacent strands and AltH1 folding requires interaction between noncontiguous regions of the RNA (Figure 5A). In HPC28-510 and HPC28-310 RNAs, H1 helices, with eight base pairs, and AltH1 helices, with 10 base pairs, have similar sequences and calculated thermodynamic stabilities as the variants examined previously in which folding of both 5′ and 3′ AltH1 structures completely inhibited intracellular ribozyme assembly (Table 1) [32].

Circularly permuted variants were equally functional during co-transcriptional assembly in vitro (Figure 5B). Chimeric HPC28-510 and HPC28-310 mRNAs containing circularly permuted ribozyme sequences with inserts located upstream or downstream of the ribozyme were less abundant in yeast relative to their mutationally inactivated control mRNAs and displayed the accelerated decay kinetics indicative of efficient intracellular self-cleavage (Figure 5C). H1 sequences in HPC28-310 mRNAs also resisted DMS modification in vivo, consistent with the assembly of functional ribozyme structures (Figure S4). The high self-cleavage activity of circular permutants suggests that H1 helices that fold from contiguous sequences, and are expected to fold rapidly, are better able to resist competition from noncontiguous AltH1 stem loops, even when AltH1 stem loops have greater thermodynamic stability.

### Crowding Agents Did Not Alter Secondary Structure Partitioning In Vitro

**Figure 6:**
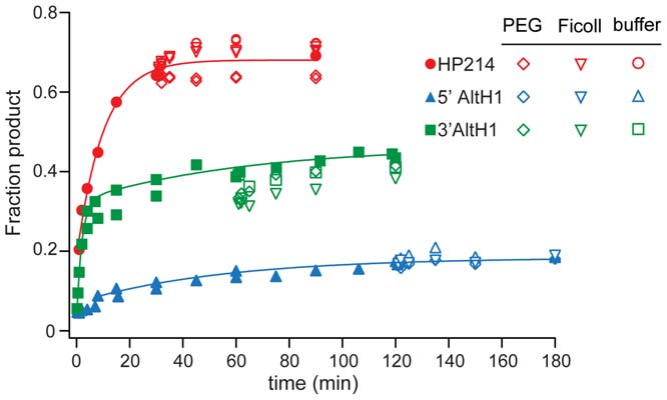
“Crowding” agents do not promote exchange among alternative RNA secondary structures in vitro. Dilution of HP214 ribozyme variants assembled during in vitro transcription into transcription buffer with 20% PEG 8000 or 20% Ficoll 400 had no significant effect on folding outcomes. Plots show results from a single representative experiment. Reported values represent the mean and standard deviation obtained from two or more experiments.

An intracellular environment contains high concentrations of macromolecules, described as “molecular crowding” [41], that might influence the stability of RNA structures [42]–[45]. We investigated the effect of molecular crowding on RNA secondary structure exchange by combining the products of co-transcriptional folding reactions with PEG or Ficoll, two crowding agents that are believed to mimic molecular crowding in vitro (Figure 6). If crowding agents lower the activation barrier to exchange between otherwise stable RNA secondary structures, HP214-512 RNAs that are kinetically trapped in a 5′ AltH1 structure that has lower thermodynamic stability than the downstream H1 helix would be expected to exchange rapidly into the thermodynamically favored ribozyme structure and self-cleave. However, we observed no change in cleavage extents for any of the HP214 variants after dilution of co-transcriptional folding reactions into high concentrations of PEG or Ficoll.

## Discussion

### mRNA Secondary Structures Fold Sequentially In Vivo

We have examined the folding behavior of chimeric mRNAs with the potential to adopt defined alternative secondary structures during co-transcriptional folding reactions in vitro and in living cells. We previously found that folding patterns were consistent with a sequential mechanism in which stable upstream structures dominate the folding outcome during co-transcriptional folding in vitro but the most thermodynamically stable structures dominated folding outcomes during assembly of the same chimeric mRNAs in yeast [32]. The current experiments were designed to probe contributions of folding kinetics and dissociation kinetics to intracellular RNA assembly and to determine whether RNA secondary structures can ever be sufficiently stable to resist thermodynamic equilibration in vivo. The folding behavior of the RNAs with extremely stable secondary structures examined here revealed that there is a threshold where intracellular secondary structure folding does occur sequentially in vivo and does not reach thermodynamic equilibrium. Furthermore, the threshold for exchange is higher and the rate of exchange between alternative secondary structures is much faster in vivo than it is in vitro.

The H1 helix of HP210-510 and HP210-310 has greater thermodynamic stability than the H1 helix with eight base pairs in the chimeric mRNAs that we examined previously by about 2 kcal/mol and is expected to dissociate more than 20-fold more slowly [32]. Functional ribozymes with 10 base pairs in H1 were able to resist competition from the downstream 3′ AltH1 in HP210-310, which has a thermodynamic advantage of −3 kcal/mol. Likewise, HP214-512 mRNAs exhibited no detectable intracellular cleavage activity even though an H1 helix with 14 base pairs has a thermodynamic advantage of −3.2 kcal/mol relative to an upstream 5′ AltH1 helix with 12 base pairs. These results suggest that these upstream 5′ AltH1 and downstream H1 structures formed sequentially during transcription and remain folded despite the potential to form alternative structures with greater thermodynamic stability by interacting with downstream sequences.

HP210-310 and HP210-312 mRNAs chimeric mRNAs share the same H1 structure with 10 base pairs but the 10- and 12-base-pair 3′ AltH1 structures differ in thermodynamic stability by 5 kcal/mol. If partitioning between H1 and 3′ AltH1 structures reflected their relative thermodynamic stabilities, the functional form of HP210-312 mRNA would have been more than 50-fold more abundant than the functional form of HP210-310 mRNA. The observation that both chimeric mRNAs exhibit similar intracellular self-cleavage kinetics suggests that folding outcomes are determined by slow H1 dissociation kinetics and not by thermodynamic equilibration.

### Rapid Folding Kinetics Might Favor Local Secondary Structures

Stem loop folding rates decrease linearly with increasing loop size in vitro [39], so folding of nonfunctional AltH1 structures from complementary strands separated by four nucleotides could have a kinetic advantage relative to H1 helices that fold from noncontiguous strands at opposite ends of the ribozyme sequence that are separated by 63 nucleotides. In the first set of ribozyme variants we examined, AltH1 stem loops folded from contiguous sequences while the H1 stem loops folded from sequences at opposite ends of the ribozyme [32]. If AltH1 structures fold first and dissociate slowly, the ability of the downstream 3′ AltH1 stem loops with moderate thermodynamic stability to inhibit ribozyme assembly could have reflected the importance of folding kinetics in folding outcomes. Indeed, chimeric mRNAs with circularly permuted ribozymes in which eight-base-pair H1 stem loops folded from contiguous strands were able to resist competition from noncontiguous AltH1 stem loops, even when upstream and downstream AltH1 stem loops had greater thermodynamic stability. Thus, contiguity might influence folding outcomes by conferring a kinetic advantage on local secondary structures.

### Rapid Exchange Between Adjacent Helices Might Occur through Branch Migration In Vivo

**Figure 7:**
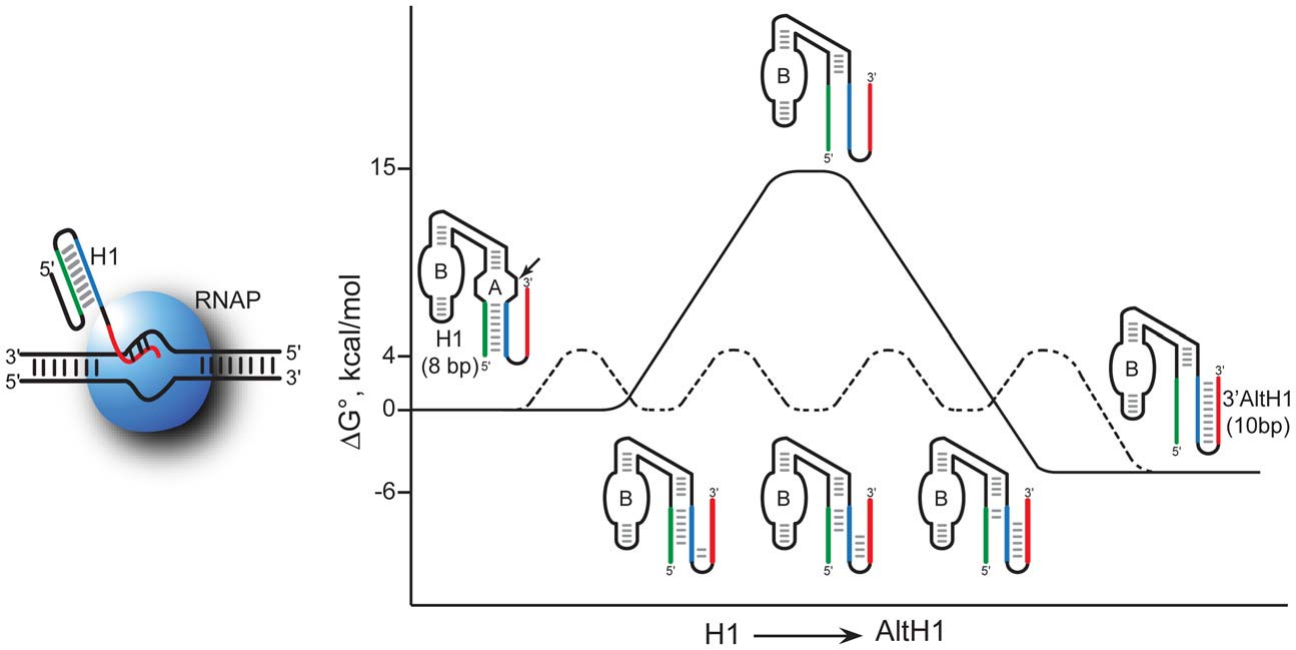
Free energy diagram of secondary structure exchange through branch migration. Exchange between adjacent RNA secondary structures occurs much faster than simple helix dissociation in vivo, suggesting that exchange occurs through a branch migration mechanism. Stepwise exchange with incremental steps encounters smaller energy barriers than two-step dissociation of a long, stable helix. Rapid exchange between adjacent helices was not observed during co-transcriptional assembly in vitro, suggesting that a component of the intracellular folding reaction, such as proteins deposited on nascent transcripts, facilitates branch migration in vivo.

Our previous studies revealed that kinetic and equilibrium parameters for intermolecular and intramolecular ribozyme reactions in yeast agree remarkably well with the same parameters measured in vitro provided that in vitro reactions approximate an intracellular ionic environment [34],[36],[38],[46]–[48]. The 5′ and 3′ products of HP self-cleavage associate through intermolecular base pairs in H1 so product dissociation kinetics reflect H1 dissociation. Cleavage products that associate through an intermolecular H1 helix with six base pairs exhibited no detectable product dissociation in vivo, and the dissociation rate constant of about 3 min^−1^ measured for a complex with four base pairs in H1 in vivo agreed remarkably well with the rate constant expected for dissociation of the same cleavage products in vitro [38]. Thus, intracellular product dissociation kinetics provided no evidence that any component of the intracellular environment significantly altered H1 stability in ribozymes without the potential to form AltH1 structures. The slow dissociation rate of an H1 helix with four base pairs is difficult to reconcile with a rapid conformational exchange model in which nonspecific RNA chaperones act generally to destabilize all RNA helices in vivo. Free energy calculations suggest that a helix with eight base pairs should dissociate about 160-fold more slowly than a helix with four base pairs but an eight-base-pair H1 helix seemed to exchange rapidly with an adjacent AltH1 helix in vivo [32]. This contrast between slow kinetics of simple helix dissociation and rapid exchange between adjacent secondary structures suggests that the intracellular mechanisms of exchange between adjacent secondary structures and simple helix dissociation are qualitatively different. Folding of a 10 base pair AltH1 during stepwise dissociation of an eight base pair H1 might facilitate exchange between neighboring structures without incurring the large energy cost required for complete dissociation of a long, stable helix. Exchange between adjacent structures might occur much faster than simple helix dissociation if RNA secondary structures exchange through a branch migration mechanism (Figure 7).

A branch migration mechanism previously was proposed to explain the lower-than-expected activation barrier observed for exchange between alternate secondary structures of a spliced leader RNA in vitro [49]. However, stable upstream 5′ AltH1 structures consistently and effectively inhibited assembly of downstream ribozymes during co-transcriptional assembly in vitro, so rapid exchange between adjacent helices was not a spontaneous process under our in vitro transcription conditions. An intracellular environment contains high concentrations of macromolecules, described as “molecular crowding” [41],[44], which is thought to influence the stability of nucleic acid structures through effects on the activity of water that modulate hydration states [42],[43],[45],[50]–[52]. However, we found no evidence of secondary structure rearrangements even when PEG or Ficoll were added to mimic crowding effects in vitro. Rapid exchange between adjacent secondary structures during co-transcriptional assembly in vivo might be explained by the ability of proteins associated with nascent transcripts to facilitate branch migration. Further work will be needed to identify which protein(s) might modulate exchange kinetics in vivo and learn whether transcripts produced by different RNA polymerases, by different forms of RNA polymerase II, or in different physiological states, exhibit different exchange kinetics.

### Implications for RNA Secondary Structure Exchange in Biological Processes

Many RNA processing and assembly events occur co-transcriptionally in vivo [53]–[58]. The H1 and AltH1 structures examined here, with free energies ranging from −15 to −25 kcal/mol, are similar in thermodynamic stability to secondary structure elements found in internal ribosome entry sites, iron response elements, selenocysteine insertion sites, histone stem loop structures, and structures implicated in alternative mRNA splicing that are found in eukaryotic mRNAs, and the secondary structures that regulate transcription and translation in the 5′ UTRs of bacterial mRNAs (Figure 1A) [3],[5],[59]–[65]. Therefore, these quantitative relationships between the thermodynamic stability of RNA secondary structures and intracellular secondary structure folding and exchange kinetics during transcription are likely to have important implications for understanding the mechanisms of RNA and RNP assembly and RNA-mediated processes in biological systems.

Some of the most detailed studies of RNA secondary structure folding and exchange mechanisms have been carried out with an adenine-responsive riboswitch found in the 5′ UTR of an mRNA in *Bacillus subtilis* that encodes a purine efflux pump [3],[5],[66]–[68]. This riboswitch consists of an upstream aptamer domain that binds adenine and a downstream expression platform domain that has the potential to terminate transcription before RNA polymerase reaches the coding region. Adenine binding to the aptamer domain affects partitioning between aptamer and terminator structures to provide feedback regulation of an adenine biosynthetic gene in response to intracellular adenine concentrations. Part of the riboswitch sequence has the potential to participate in mutually exclusive aptamer or transcription termination structures (Figure 1A), similar to the chimeric mRNAs examined here, depending on whether adenine is bound. In a rapid exchange model of the switch mechanism, bound and unbound conformations are in rapid exchange and ligand binding drives folding of the ligand-bound structure by increasing its thermodynamic stability. With the free energies of the aptamer and terminator structures on the order of −12 and −32 kcal/mol, respectively [67], exchange between alternative structures could be too slow for adenine binding to drive conversion from the terminator to the aptamer structure on a biologically relevant time scale. Indeed, results of careful bulk and single molecule analyses of folding, ligand binding and transcription elongation kinetics of an adenine-responsive riboswitch in vitro argue against a thermodynamic equilibration model of riboswitch activation and point to a kinetically controlled process in which ligand binding to the nascent transcript stabilizes the bound aptamer conformation before transcription and assembly of the downstream transcription termination sequence is complete. Kinetic control of partitioning between alternative secondary structures has also been proposed in regulation of translation initiation and viral replication, for example [28],[69],[70].

It is not clear yet how predictions based on riboswitch folding behavior in vitro relate to metabolite-regulation of gene expression in vivo. Chimeric mRNAs that have competing secondary structures with nine or fewer base pairs and free energies above −15 kcal/mol appeared to exchange rapidly in vivo (Table 1) [32]. However, chimeric mRNAs became kinetically trapped in upstream secondary structures with free energies ranging from −17 to −25 kcal/mol even when downstream sequences had the potential to form alternative structures that were more stable by 3 to 6 kcal/mol (Table 1). These results delineate a very narrow threshold of thermodynamic stability that determines whether thermodynamics or folding and unfolding rates govern the folding outcome for a particular mRNA. This narrow range of free energy over which folding outcomes reflect thermodynamic equilibria or the kinetics of folding and unfolding suggests an elegant mechanism for regulating a switch through ligand binding. In the case of the adenine riboswitch, for example, adenine binding was found to stabilize an adenine aptamer structure by about 4 kcal/mol [68]. In our yeast mRNA system, a decrease in free energy from −12 to −16 kcal/mol would be sufficient to shift from a rapid to a slow exchange folding mechanism. It is important to note that our studies address secondary structure exchange in eukaryotic mRNAs transcribed by RNA Pol II in yeast, but similar RNA switches also are likely to participate in eukaryotic gene regulation. Further studies will be needed to establish whether the free energy threshold that distinguishes between rapid and slow exchange regimes varies among different biological systems. Nonetheless, it is intriguing to speculate that RNA binding proteins or small molecules like adenine with equilibrium dissociation constants in the millimolar range could provide more than enough stabilizing energy to drive an RNA secondary structure across this threshold and kinetically trap a specific ligand-bound secondary structure in vivo.

## Materials and Methods

### Plasmid Construction and Propagation

Plasmid templates for in vitro transcription were derived from pTLR28, a pUC18 variant in which ribozyme-coding sequences are fused to a T7 RNA polymerase promoter [46]. Sequence changes were introduced using QuikChange™ mutagenesis (Stratagene) and the primers shown in Table S1. To construct plasmids for T7 RNA polymerase transcription of circularly permuted ribozymes in vitro, a DNA fragment that encodes a circularly permuted ribozyme fused to a T7 RNA polymerase promoter was obtained by overlapping polymerase chain reaction (PCR) using the four oligonucleotides listed in Table S1, and the fragment was digested with Kpn I and EcoR I and inserted into the pUC18 polylinker. Further changes to helix and loop sequences were performed using QuikChange™ mutagenesis and the primers shown in Table S1.

For expression of chimeric mRNAs in yeast, sequences encoding ribozyme variants were inserted into the 3′ UTR of the yeast PGK1 gene in pGAL28, a pRS316 derivative in which the PGK1 gene is fused to the GAL1 upstream activation sequence [36],[38],[71]. The unique Cla I site in the 3′ UTR of PGK1 was replaced with Mlu I and Afl II sites, the same sites were introduced at the opposite end of the ribozyme sequences in pUC18 derivatives using primers shown in Table S1, and the Mlu I Afl II fragments were ligated to produce pGAL28 derivatives.

Ribozyme names reflect the nature of the interdomain junction (two-way), the number of base pairs in H1, the location of a complementary insertion (5′ or 3′), and the number of base pairs in AltH1. For example, HP210-510 is a HP with a two-way helical junction that has 10 base pairs in H1 and a complementary insert located on the 5′ side of the ribozyme with the potential to form a 5′ AltH1 stem loop that has 10 base pairs. “m” indicates the presence of an inactivating G+1A mutation. Plasmids were propagated in *Escherichia coli* strain DH5α [72] or XL-Blue (Stratagene) or in *S. cerevisiae* strain HFY114 (*MATa ade2-1 his3-11,15 leu2-3 112 trp1-1 ura3-1 can1-100*) [73].

### Assembly and Self-Cleavage Kinetics In Vitro

Co-transcriptional self-cleavage kinetics were measured in vitro as described [32],[74]. Briefly, linearized plasmid template DNA, at a concentration of 29 nM, was pre-incubated at 30°C for 10 min in 30 mM HEPES (pH 7.8 at 30°C), 1 mM DTT, 0.25 mM EDTA, 96 mM sodium glutamate and either 16 mM magnesium acetate, along with 4 mM of each NTP, 1µL of RNAsin (40U/µL, Promega), and 10–40 µCi [α-^32^P] ATP (3,000 Ci/mmole, NEN) in a volume of 57 µL, as described [75], except for the change in magnesium concentration. To start the reaction, 3 µL of T7 RNA polymerase, freshly prepared at a concentration of 0.2 mg/mL in transcription buffer with 1% Tween 20 (Sigma), was added for a final reaction volume of 60 µL with 0.01 mg/mL T7 RNA polymerase and 0.05% Tween 20. Aliquots were removed at intervals over 2 h, quenched by the addition of gel loading buffer (90% formamide, 25 mM EDTA, 0.002% xylene cyanole, and 0.002% bromophenol blue) and fractionated by denaturing gel electrophoresis. In every case, care was taken to ensure that transcription rates remained linear throughout each time course so that self-cleavage rates could be computed accurately from the fit to single exponential or double exponential rate equations [74]. The plots shown in the figures represent the results of a single representative experiment. Reported values represent the mean and standard deviation obtained from two or more experiments. In experiments designed to measure the effects of crowding agents, transcription reactions were diluted 2-fold into transcription buffer containing either 40% PEG 8000 or 40% Ficoll 400 and incubated at 30°C for up to 1 h before aliquots were combined with gel loading buffer.

### Intracellular Folding and Self-Cleavage Kinetics

For intracellular self-cleavage assays, RNA was extracted from log phase yeast cultures grown at 30°C in minimal medium after the addition of glucose to inhibit transcription and quantified using RNase protection assays, as described [32],[33]. The ^32^P-labeled RNAs used as hybridization probes were transcribed from linearized pGEM-4Z derivatives (Promega), as described [38]. When ^32^P-labeled self-cleaving RNA was combined with yeast pellets and subjected to extraction and analysis procedures, in control experiments that were carried out in parallel with every assay, less than 10% of uncleaved ribozyme RNAs underwent cleavage, confirming that conditions used for RNase protection assays do not support ribozyme activity. Intracellular chimeric mRNA decay rates were calculated by fitting to a single exponential rate equation. Intracellular self-cleavage rates were calculated from the difference between decay rates of uncut self-cleaving mRNAs and chimeric mRNAs with an inactivating G+1A mutation as described [33]. Uncut HP mRNA abundance was normalized by comparison with *ACT1* mRNA. The plots shown in the figures represent the results of a single representative decay time course experiment. Reported values represent the mean and standard deviation obtained from two or more experiments. All mutationally inactivated chimeric mRNAs displayed the same degradation rate of 0.043±0.003 m^−1^. At steady state, self-cleaving mRNAs are present at lower levels than mutationally activated chimeric mRNAs because both kinds of chimeric mRNAs are synthesized at the same rate, but self-cleaving RNAs decay both through self-cleavage and through normal mRNA degradation pathways while mutationally inactivated RNAs only decay through intrinsic degradation pathways. Therefore, intracellular cleavage rates also can be calculated from the relative abundance of self-cleaving and mutationally inactivated chimeric mRNAs at steady state when the intrinsic degradation rate is known [33]. Intracellular cleavage rates determined using both methods typically agree within 30% and never vary more than 2-fold.

### Chemical Structure Mapping

Chemical structure mapping was used to confirm that nonfunctional structures contained AltH1 and not H1 structures, as expected (Figures S1, S2, S3, S4). DMS modification of intracellular yeast RNA was performed essentially as described [76] using chimeric mRNAs that contained the inactivating G+1A mutation [33]. Yeast at mid-log phase were pelleted and resuspended in 1/50 vol minimal medium, combined with 4 µL DMS and allowed to react at 30°C for 2 min with frequent mixing. The modification reaction was quenched with 25 µL ß-mercaptoethanol, then yeast were washed by vigorous mixing with 0.25 mL of ice-cold 0.7 M ßME, pelleted, and then washed with 1 mL ice-cold water.

DMS modified adenosine and cytosine residues were identified as blocks to reverse transcription [77]. For primer extension reactions, 20 µg of yeast RNA and 0.2 pmole of [5′-^32^P] PX4 primer were annealed in 3 µl 50 mM Tris Cl (pH 8.3 at 42°C), 0.1 mM EDTA by heating to 95°C and cooling to 50°C over 45 min, then adjusted to 50 mM Tris Cl pH 8.3, 50 mM KCl, 10 mM MgCl_2_, 10 mM DTT, 0.5 mM spermidine, 0.5 mM each dNTP, and 0.17 units/µl AMV reverse transcriptase in 6 µl, and incubated at 50°C for 45 min. Parallel sequencing reactions also contained 0.5 mM ddATP, ddCTP, ddGTP, or ddTTP. Reaction products were fractionated by gel electrophoresis and quantified through radioanalytic imaging. Profiles represent the relative amounts of primer extension products after normalization to the intensity of the band corresponding to unmodified uridine at position U+5 of the ribozyme unless otherwise indicated.

## Supporting Information

Figure S1 **Chemical protection mapping of HP structures assembled in yeast.** Nucleotide bases that were accessible to modification by DMS are indicated by orange circles. Adenine and cytosine residues engaged in interactions with complementary bases or possible proteins are expected to resist methylation by DMS. Intensities were normalized relative to the band corresponding to the unmodified uridine at position 5 of the ribozyme.
(2.55 MB TIF)

Figure S2 **Chemical protection mapping of HP structures assembled in yeast.** Nucleotide bases that were accessible to modification by DMS are indicated by orange circles. Adenine and cytosine residues engaged in interactions with complementary bases or possible proteins are expected to resist methylation by DMS. Intensities were normalized relative to the band corresponding to the unmodified uridine at position 5 of the ribozyme.
(2.91 MB TIF)

Figure S3 **Chemical protection mapping of HP structures assembled in yeast.** Nucleotide bases that were accessible to modification by DMS are indicated by orange circles. Adenine and cytosine residues engaged in interactions with complementary bases or possible proteins are expected to resist methylation by DMS. Intensities were normalized relative to the band corresponding to the unmodified uridine at position 5 of the ribozyme.
(2.89 MB TIF)

Figure S4 **Chemical protection mapping of HP structures assembled in yeast.** Nucleotide bases that were accessible to modification by DMS are indicated by orange circles. Adenine and cytosine residues engaged in interactions with complementary bases or possible proteins are expected to resist methylation by DMS. Intensities were normalized relative to the band corresponding to the unmodified guanine at position +10 of the ribozyme for HPC28, an unmodified uridine at position +9 for HPC28-510, and an unmodified uridine at position +8 for HPC28-310.
(1.95 MB TIF)

Table S1 **Oligonucleotides used for plasmid constructions.** Plasmids were constructed using conventional procedures as described in Materials and Methods.
(0.12 MB DOC)

## Abbreviations

AltH1: alternative helix 1
DMS: dimethyl sulfate
H1: helix 1
HP: hairpin ribozyme
PCR: polymerase chain reaction
UTR: untranslated region
