# Supplementary figures and images for: mRNA Secondary Structures Fold Sequentially But Exchange Rapidly In Vivo
Source: PLoS Biol. 2010 Feb 9;8(2):e1000307. doi: 10.1371/journal.pbio.1000307 (PMC2817708; doi:10.1371/journal.pbio.1000307)

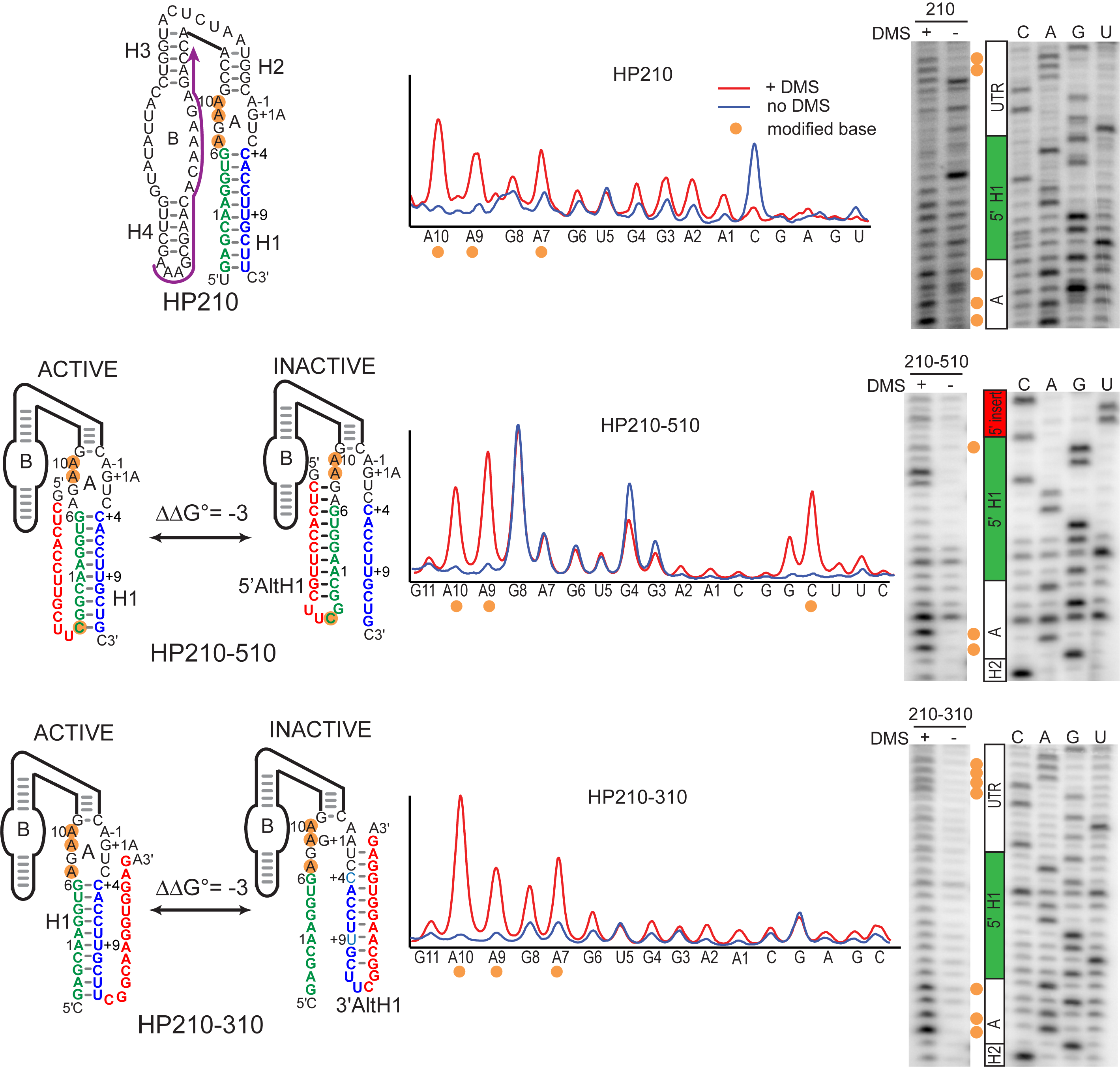

Supplement: Figure S1 — Chemical protection mapping of HP structures assembled in yeast. Nucleotide bases that were accessible to modification by DMS are indicated by orange circles. Adenine and cytosine residues engaged in interactions with complementary bases or possible proteins are expected to resist methylation by DMS. Intensities were normalized relative to the band corresponding to the unmodified uridine at position 5 of the ribozyme. (2.55 MB TIF) [file pbio.1000307.s001.tif]

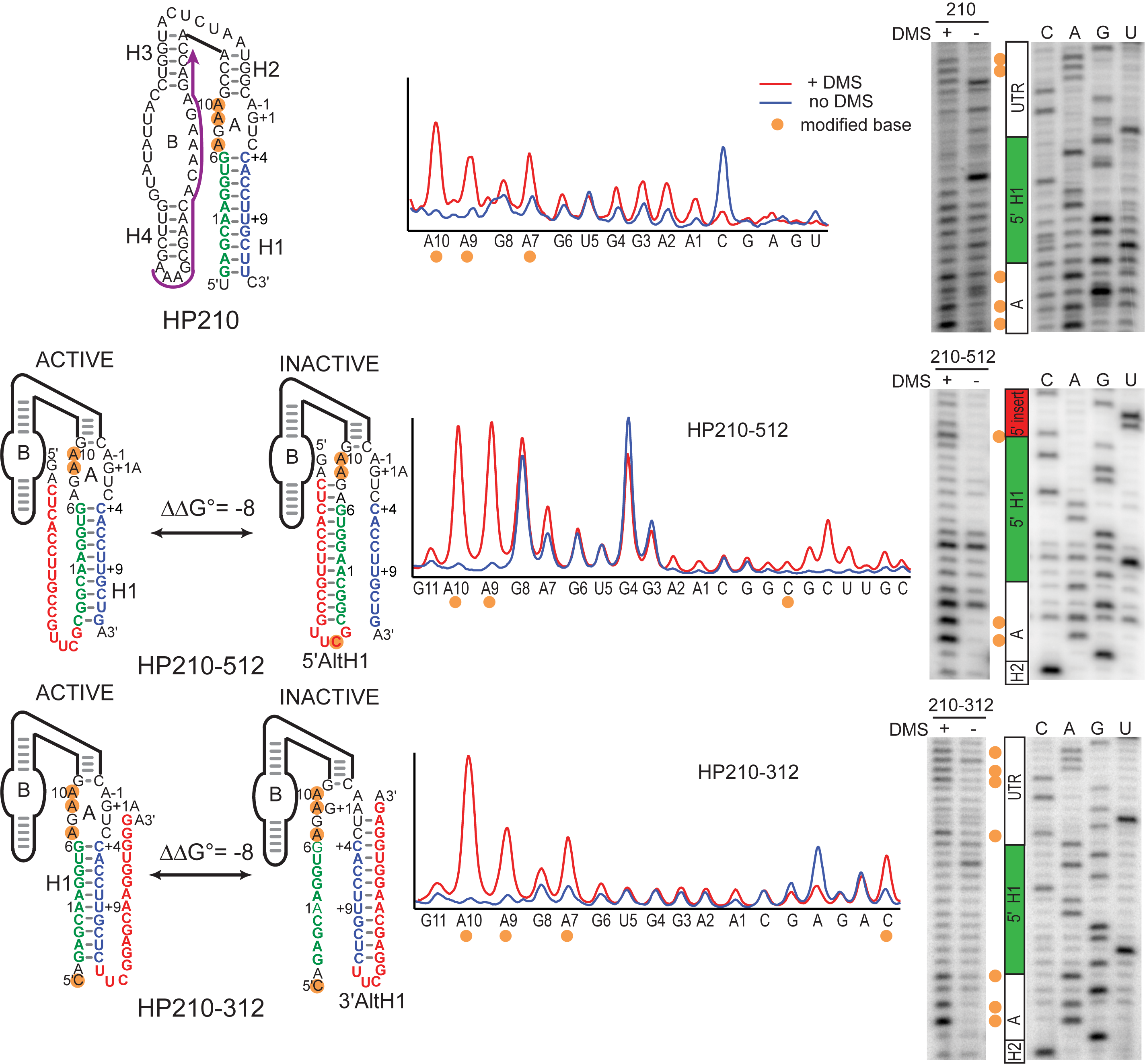

Supplement: Figure S2 — Chemical protection mapping of HP structures assembled in yeast. Nucleotide bases that were accessible to modification by DMS are indicated by orange circles. Adenine and cytosine residues engaged in interactions with complementary bases or possible proteins are expected to resist methylation by DMS. Intensities were normalized relative to the band corresponding to the unmodified uridine at position 5 of the ribozyme. (2.91 MB TIF) [file pbio.1000307.s002.tif]

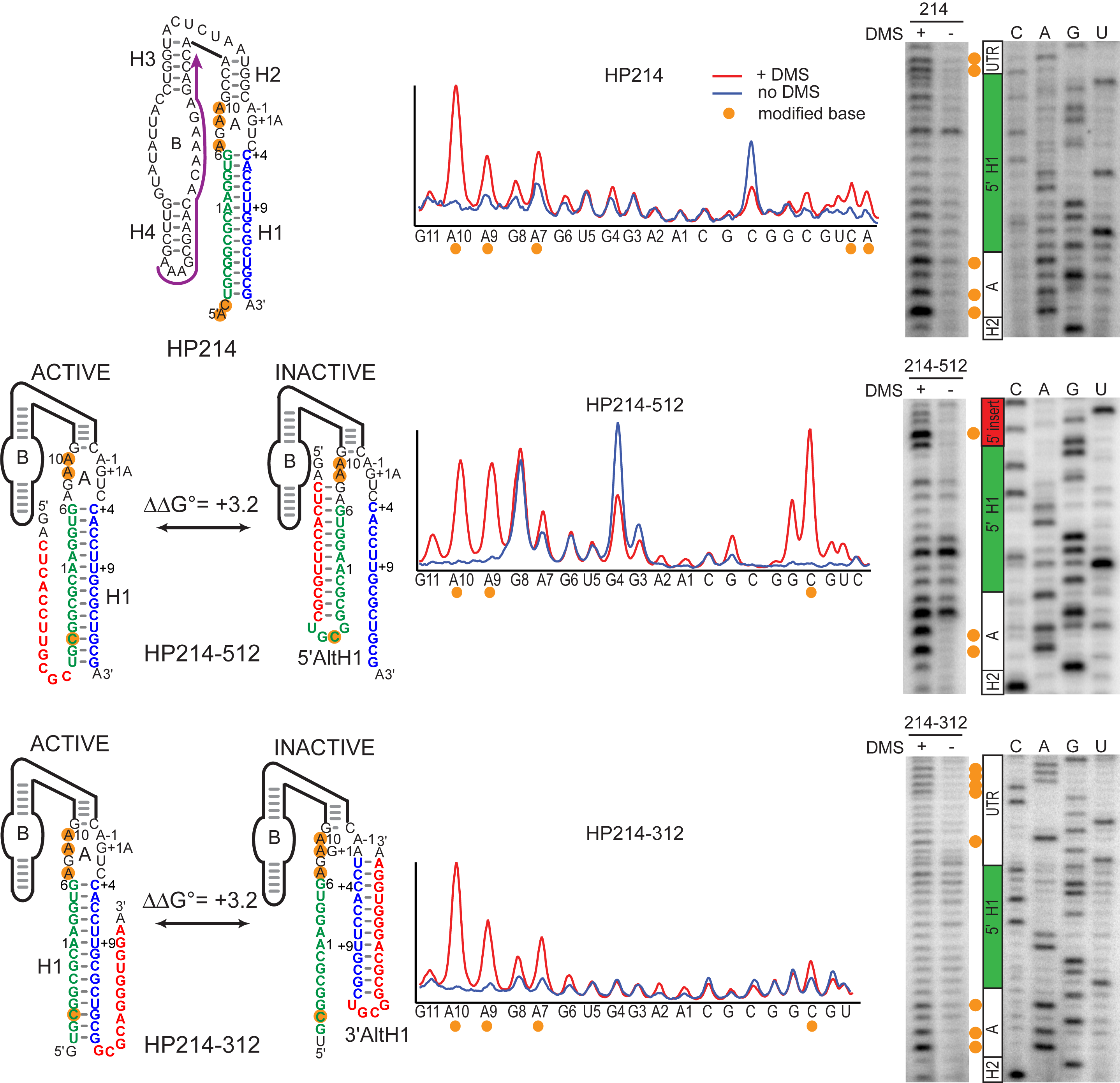

Supplement: Figure S3 — Chemical protection mapping of HP structures assembled in yeast. Nucleotide bases that were accessible to modification by DMS are indicated by orange circles. Adenine and cytosine residues engaged in interactions with complementary bases or possible proteins are expected to resist methylation by DMS. Intensities were normalized relative to the band corresponding to the unmodified uridine at position 5 of the ribozyme. (2.89 MB TIF) [file pbio.1000307.s003.tif]

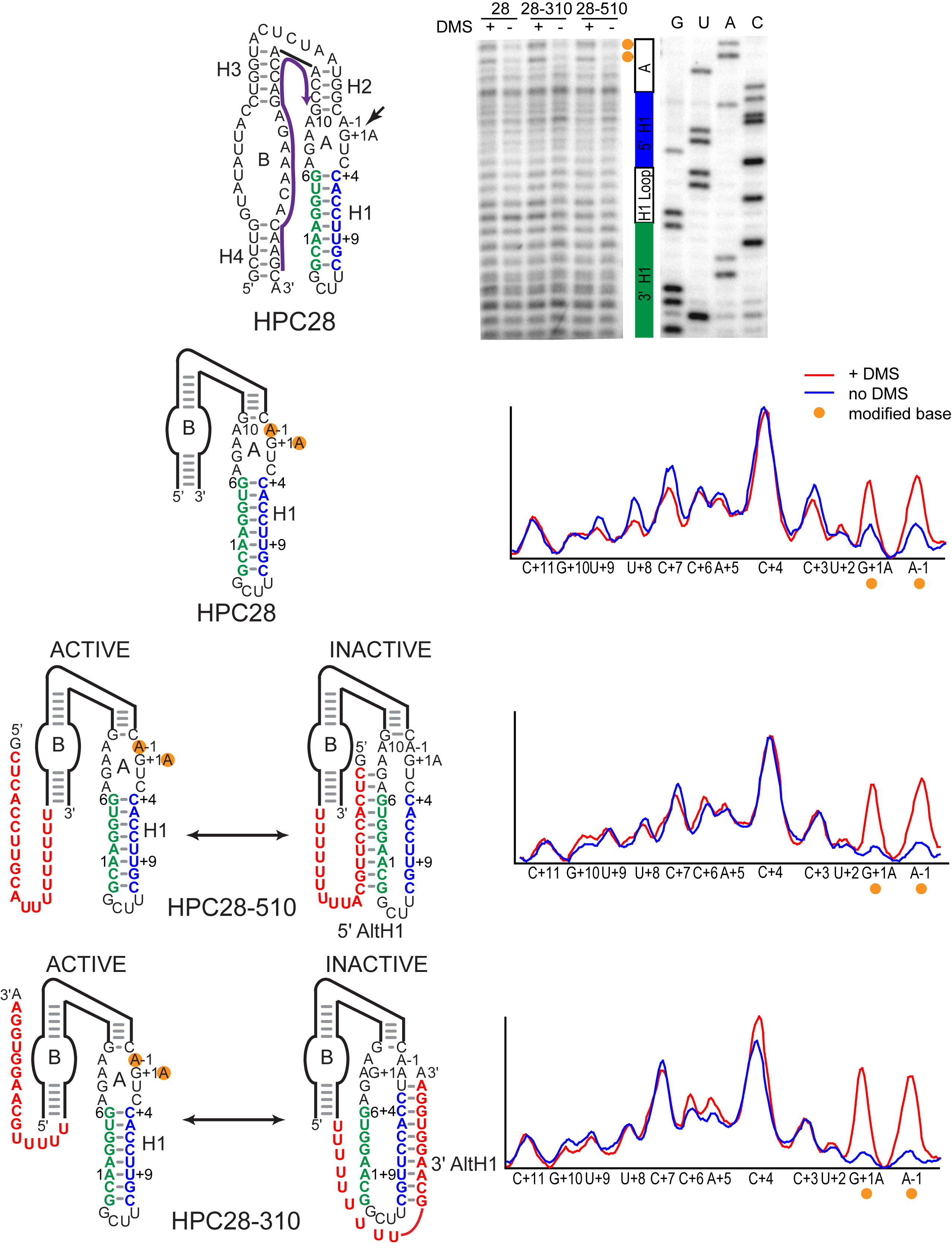

Supplement: Figure S4 — Chemical protection mapping of HP structures assembled in yeast. Nucleotide bases that were accessible to modification by DMS are indicated by orange circles. Adenine and cytosine residues engaged in interactions with complementary bases or possible proteins are expected to resist methylation by DMS. Intensities were normalized relative to the band corresponding to the unmodified guanine at position +10 of the ribozyme for HPC28, an unmodified uridine at position +9 for HPC28-510, and an unmodified uridine at position +8 for HPC28-310. (1.95 MB TIF) [file pbio.1000307.s004.tif]
