# Supplementary material for: mRNA Secondary Structures Fold Sequentially But Exchange Rapidly In Vivo
Source: PLoS Biol. 2010 Feb 9;8(2):e1000307. doi: 10.1371/journal.pbio.1000307 (PMC2817708; doi:10.1371/journal.pbio.1000307)
Supplement: Table S1 — Oligonucleotides used for plasmid constructions. Plasmids were constructed using conventional procedures as described in Materials and Methods. (0.12 MB DOC) [file pbio.1000307.s005.doc]

**Supplemental Table 1**. Oligonucleotides used for plasmid constructions

| Plasmid | Ribozyme | Primer | Sense/Antisense |
| --- | --- | --- | --- |
| pT210 | HP210 | 210B5F | 5'CGACTCACTATAGAGCAAGGTGAGAAGCC |
|  |  | 210B5R | 5'GGCTTCTCACCTTGCTCTATAGTGAGTCG |
|  |  | 210B3F | 5'GGCAGTCCACCTTGCTTCTTAAGAGATCTGGTACCGAGC |
|  |  | 210B3R | 5'GCTCGGTACCAGATCTCTTAAGAAGCAAGGTGGACTGCC |
| pT210-510 | HP210-510 | 210B5105F | 5'CCCCGGGTACCTAATACGACTCACTATAGCTCACCTTGCTTCGGCAAGGTGAGAAGCCAACCAGAGAAACAC  AAGCG |
|  |  | 210B5105R | 5'CGCTTGTGTTTCTCTGGTTGGCTTCTCACCTTGCCGAAGCAAGGTGAGCTATAGTGAGTCGTATTAGGTACCC  GGGG |
|  |  | 210B5103F | 5'CCACCTTGCTGCTTAAGAGATCTGG |
|  |  | 210B5103R | 5'CCAGATCTCTTAAGCAGCAAGGTGG |
| pT210-310 | HP210-310 | 210B3105F | 5'CCTAATACGACTCACTATAGCGAGCAAGGTGAGAAGCC |
|  |  | 210B3105R | 5'GGCTTCTCACCTTGCTCGCTATAGTGAGTCGTATTAGG |
|  |  | 210B3103F | 5'GGTACTCTAATGGCAGTCCACCTTGCTTCGGCAAGGTGGAGCTTAAGAGATCTGGTACCGAGCTCGAATTCG |
|  |  | 210B3103R | 5'CGAATTCGAGCTCGGTACCAGATCTCTTAAGCTCCACCTTGCCGAAGCAAGGTGGACTGCCATTAGAGTACC |
| pUCHP210 | HP210 | 210BMLUF | 5'CCTAATACGACTCACTATAACGCGTGAGCAAGGTGAGAAGCC |
|  |  | 210BMLUR | 5'GGCTTCTCACCTTGCTCACGCGTTATAGTGAGTCGTATTAGG |
| pUCHP210-510 | HP210-510 | LR210510MLUF | 5'CCTAATACGACTCACTATAACGCGTGCTCACCTTGCTTCGGC |
|  |  | LR210510MLUR | 5'GCCGAAGCAAGGTGAGCACGCGTTATAGTGAGTCGTATTAGG |
| pUCHP210-310 | HP210-  310 Insert | 210B310MLUF | 5'CCTAATACGACTCACTATAACGCGTGCGAGCAAGGTGAGAAGCC |
|  |  | 210B310MLUR | 5'GGCTTCTCACCTTGCTCGCACGCGTTATAGTGAGTCGTATTAGG |
| pT210-512 | HP210-512 | 210B5125F | 5'GGGTACCTAATACGACTCACTATAGACTCACCTTGCCGTTCGCGGCAAGGTGAGAAGCCAACCAGAGAAACAC  AAGCG |
|  |  | 210B5125R | 5'CGCTTGTGTTTCTCTGGTTGGCTTCTCACCTTGCCGCGAACGGCAAGGTGAGTCTATAGTGAGTCGTATTAGGTACCC |
|  |  | 210B5123F | 5'CCACCTTGCTGACTTAAGAGATCTGG |
|  |  | 210B5123R | 5'CCAGATCTCTTAAGTCAGCAAGGTGG |
| pT210-312 | HP210-312 | 210B3125F | 5'CCTAATACGACTCACTATAGCAGAGCAAGGTGAGAAGCC |
|  |  | 210B3125R | 5'GGCTTCTCACCTTGCTCTGCTATAGTGAGTCGTATTAGG |
|  |  | 210B3123F | 5'GGTACTCTAATGGCAGTCCACCTTGCTCTTCGGAGCAAGGTGGGACTTAAGAGATCTGGTACCGAGCTCGAATT  CG |
|  |  | 210B3123R | 5'CGAATTCGAGCTCGGTACCAGATCTCTTAAGTCCCACCTTGCTCCGAAGAGCAAGGTGGACTGCCATTAGAGT  ACC |
| pT214 | HP214 | 214AF | 5'CGACTCACTATAGACTGCGGCGCAAGGTGAGAAGCC |
|  |  | 214AR | 5'GGCTTCTCACCTTGCGCCGCAGTCTATAGTGAGTCG |
|  |  | 214BF | 5'GGCAGTCCACCTTGCGCTGCGACTTAAGAGATCTGGTACCG |
|  |  | 214BR | 5'CGGTACCAGATCTCTTAAGTCGCAGCGCAAGGTGGACTGCC |
| pP214 | HP214 | p214AF | 5'GAATTGAAACGCGTGACTGCGGCGCAAGGTGAGAAGCC |
|  |  | p214AR | 5'GGCTTCTCACCTTGCGCCGCAGTCACGCGTTTCAATTC |
|  |  | p214BF | 5'CTAATGGCAGTCCACCTTGCGCTGCGACTTAAGAGATCAATTTTTTTCTTTTC |
|  |  | p214BR | 5'GAAAAGAAAAAAATTGATCTCTTAAGTCGCAGCGCAAGGTGGACTGCCATTAG |
| p214 | HP214 | p214AF | GAATTGAAACGCGTGACTGCGGCGCAAGGTGAGAAGCC |
|  |  | p214AR | GGCTTCTCACCTTGCGCCGCAGTCACGCGTTTCAATTC |
|  |  | p214BF | CTAATGGCAGTCCACCTTGCGCTGCGACTTAAGAGATCAATTTTTTTCTTTTC |
|  |  | p214BR | GAAAAGAAAAAAATTGATCTCTTAAGTCGCAGCGCAAGGTGGACTGCCATTAG |
| pT214-512 | HP214-512 | 214512BF | GGCAGTCCACCTTGCGCTGCGAACTTAAGAGATCTGG |
|  |  | 214512BR | CCAGATCTCTTAAGTTCGCAGCGCAAGGTGGACTGCC |
|  |  | 214512AF3A | CGACTCACTATAGACCGCTGCGGCGCAAGG |
|  |  | 214512AR3A | CCTTGCGCCGCAGCGGTCTATAGTGAGTCG |
| p214-512 | HP214-512 | p214512BF | CTAATGGCAGTCCACCTTGCGCTGCGACTTAAGAGATCAATTTTTTTCTTTTC |
|  |  | p214512BR | GAAAAGAAAAAAATTGATCTCTTAAGTCGCAGCGCAAGGTGGACTGCCATTAG |
|  |  | p214512AF3A | GAAACGCGTGACCGCTGCGGCGCAAG |
|  |  | p214512AR3A | CTTGCGCCGCAGCGGTCACGCGTTTC |
|  |  | p214512AF3B | GAAACGCGTGACTCACCTTGCGCTGCGGCG |
|  |  | p214512AR3B | CGCCGCAGCGCAAGGTGAGTCACGCGTTTC |
| pT214-312 | HP214-312 | 214312ASF | CGACTCACTATAGAGTGCGGCGCAAGGTGAGAAGCCAACC |
|  |  | 214312ASR | GGTTGGCTTCTCACCTTGCGCCGCACTCTATAGTGAGTCG |
|  |  | 214312BF3A | CCTTGCGCTGCGGCGACTTAAGAGATCTGG |
|  |  | 214312BR3A | CCAGATCTCTTAAGTCGCCGCAGCGCAAGG |
|  |  | 214312BF3B | CGCTGCGGCGCAGGGTGGAACTTAAGAGATCTGG |
|  |  | 214312BR3B | CCAGATCTCTTAAGTTCCACCCTGCGCCGCAGCG |
| pP214-312 | HP214-312 | p214312AF | GAATTGAATTGAAACGCGTGAGTGCGGCGCAAGGTGAGAAGCCAACCAG |
|  |  | p214312AR | CTGGTTGGCTTCTCACCTTGCGCCGCACTCACGCGTTTCAATTCAATTC |
|  |  | p214312BF3A | CCACCTTGCGCTGCGGCGACTTAAGAGATC |
|  |  | p214312BR3A | GATCTCTTAAGTCGCCGCAGCGCAAGGTGG |
|  |  | p214312BF3B | CGCTGCGGCGCAGGGTGGAACTTAAGAGATCAATTTTTTTC |
|  |  | p214312BR3B | GAAAAAAATTGATCTCTTAAGTTCCACCCTGCGCCGCAGCG |
| p214-312 | HP214-312 | p214312AF | GAATTGAATTGAAACGCGTGAGTGCGGCGCAAGGTGAGAAGCCAACCAG |
|  |  | p214312AR | CTGGTTGGCTTCTCACCTTGCGCCGCACTCACGCGTTTCAATTCAATTC |
|  |  | p214312BF3A | CCACCTTGCGCTGCGGCGACTTAAGAGATC |
|  |  | p214312BR3A | GATCTCTTAAGTCGCCGCAGCGCAAGGTGG |
|  |  | p214312BF3B | CGCTGCGGCGCAGGGTGGAACTTAAGAGATCAATTTTTTTC |
|  |  | p214312BR3B | GAAAAAAATTGATCTCTTAAGTTCCACCCTGCGCCGCAGCG |
| pUCHP210-512 | HP210-512 | 210B512MLUF | 5'CCTAATACGACTCACTATAACGCGTGACTCACCTTGCCGTTC |
|  |  | 210B512MLUR | 5'GAACGGCAAGGTGAGTCACGCGTTATAGTGAGTCGTATTAGG |
| pUCHP210-312 | HP210-312 | 210B312MLUF | 5'CCTAATACGACTCACTATAACGCGTGCAGAGCAAGGTGAGAAGCC |
|  |  | 210B312MLUR | 5'GGCTTCTCACCTTGCTCTGCACGCGTTATAGTGAGTCGTATTAGG |
| p2XX-YYYm | HPm | 210BMF | 5'GGTACTCTAATGGCAATCCACCTTGC |
|  |  | 210BMR | 5'GCAAGGTGGATTGCCATTAGAGTACC |
| pCLR28 | HPC28 | C1LR28-1 | 5’CTAATACGACTCACTATAGCTTGGTATATTACCTGGTACTCTAATGGCAGTCCACCTTGCG |
|  |  | C1LR28-2L | 3’AAAGCAAGGTGAGAAGCCAACCAGAGAAACACAAGCAGATCTGGTACCGAGCTCG |
|  |  | C1LR28-3 | 5'GGTAATATACCAAGCTATAGTGAGTCGTATTAGGTAC |
|  |  | C1LR28-4L | 5'AATTCGAGCTCGGTACCAGATCTGCTTGTGTTTCTCTGGTTGGCTTCTCACCTTGCTTTCGCAAGGTGGACTGCCA  TTAGAGTACCA |
|  |  | C28AFLF | 5'CCAGAGAAACACAAGCTTAAGAGATCTGGTACCGAGC |
|  |  | C28AFLR | 5'GCTCGGTACCAGATCTCTTAAGCTTGTGTTTCTCTGG |
|  |  | CLRH1LOOPF | 5'GGTACTCTAATGGCAGTCCACCTTGCTTCGGCAAGGTGAGAAGCCAACCAGAGAAACAC |
|  |  | CLRH1LOOPR | 5'GTGTTTCTCTGGTTGGCTTCTCACCTTGCCGAAGCAAGGTGGACTGCCATTAGAGTACC |
| pCLR28-510 | HPC28-510 | C289UA510F | 5’CCCCGGGTACCTAATACGACTCACTATAGCTCACCTTGCATTTTTTTTTGCTTGGTATATTACCTGGTACTCTAAT  GGC |
|  |  | C289UA510R | 3’GCCATTAGAGTACCAGGTAATATACCAAGCAAAAAAAAATGCAAGGTGAGCTATAGTGAGTCGTATTAGGTAC  CCGGGG |
| pCLR28-310 | HPC28-310 | C289U310F | 5’GGTGAGAAGCCAACCAGAGAAACACAAGCTTTTTTTTTGCAAGGTGGAAACTTAAGAGATCTGGTACCGAGCT  CGAATTCG |
|  |  | C289U310R | 3’CGAATTCGAGCTCGGTACCAGATCTCTTAAGTTTCCACCTTGCAAAAAAAAAGCTTGTGTTTCTCTGGTTGGCTTCT  CACC |
| pUCHPC28 | HPC28 | C28MLUF | 5'CCTAATACGACTCACTATAACGCGTGCTTGGTATATTACCTGG |
|  |  | C28MLUR | 5'CCAGGTAATATACCAAGCACGCGTTATAGTGAGTCGTATTAGG |
| pUCHPC28-510 | HPC28-510 | C28510MLUF | 5'CGACTCACTATAACGCGTGCTCACCTTGC |
|  |  | C28510MLUR | 5'GCAAGGTGAGCACGCGTTATAGTGAGTCG |
| pUCHPC28-310 | HPC128-310 | C28MLUF | 5'CCTAATACGACTCACTATAACGCGTGCTTGGTATATTACCTGG |
|  |  | C28MLUR | 5'CCAGGTAATATACCAAGCACGCGTTATAGTGAGTCGTATTAGG |
| C28G+1A |  | C28MF | 5'GGTACTCTAATGGCAATCCACCTTGCTTCGG |
|  |  | C28MR | 5'CCGAAGCAAGGTGGATTGCCATTAGAGTACC |
| p28 | HP28 | PMLUF | 5’CCGAAAAGAAATAAATTGAATTGAATTGAAACGCGTCGCAAGGTGAGAAGCCAACCAGAG |
|  |  | PMLUR | 3’CTCTGGTTGGCTTCTCACCTTGCGACGCGTTTCAATTCAATTCAATTTATTTCTTTTCGG |
|  |  | PGAL28+3’MUTF | 5'CTCTAATGGCAGTCCACCTTGCCTTAAGAGATCAATTTTTTTCTTTTCTCTTTCC |
|  |  | PGAL28+3’MUTR | 5'GGAAAGAGAAAAGAAAAAAATTGATCTCTTAAGGCAAGGTGGACTGCCATTAGAG |

**Supplemental Table 1 legend**

Plasmids were constructed using conventional procedures as described in Materials and Methods.
